# Cross platform analysis of methylation, miRNA and stem cell gene expression data in germ cell tumors highlights characteristic differences by tumor histology

**DOI:** 10.1186/s12885-015-1796-6

**Published:** 2015-10-23

**Authors:** Jenny N. Poynter, Jessica R. B. M. Bestrashniy, Kevin A. T. Silverstein, Anthony J. Hooten, Christopher Lees, Julie A. Ross, Jakub Tolar

**Affiliations:** 1 Division of Pediatric Epidemiology and Clinical Research, University of Minnesota, Minneapolis, MN 55455 USA; 2 Masonic Cancer Center, University of Minnesota, Minneapolis, MN 55455 USA; 3 Division of Pediatric Blood and Marrow Transplantation, University of Minnesota, Minneapolis, MN 55455 USA; 4 Minnesota Supercomputing Institute, University of Minnesota, Minneapolis, MN 55455 USA; 5 Stem Cell Institute, University of Minnesota, Minneapolis, MN 55455 USA; 6 Corresponding address: 420 Delaware St SE MMC 715, Minneapolis, MN 55455 USA

**Keywords:** Pediatric cancer, Germ cell tumors, miRNA, Methylation, Stem cell

## Abstract

**Background:**

Alterations in methylation patterns, miRNA expression, and stem cell protein expression occur in germ cell tumors (GCTs). Our goal is to integrate molecular data across platforms to identify molecular signatures in the three main histologic subtypes of Type I and Type II GCTs (yolk sac tumor (YST), germinoma, and teratoma).

**Methods:**

We included 39 GCTs and 7 paired adjacent tissue samples in the current analysis. Molecular data available for analysis include DNA methylation data (Illumina GoldenGate Cancer Methylation Panel I), miRNA expression (NanoString nCounter miRNA platform), and stem cell factor expression (SABiosciences Human Embryonic Stem Cell Array). We evaluated the cross platform correlations of the data features using the Maximum Information Coefficient (MIC).

**Results:**

In analyses of individual datasets, differences were observed by tumor histology. Germinomas had higher expression of transcription factors maintaining stemness, while YSTs had higher expression of cytokines, endoderm and endothelial markers. We also observed differences in miRNA expression, with miR-371-5p, miR-122, miR-302a, miR-302d, and miR-373 showing elevated expression in one or more histologic subtypes. Using the MIC, we identified correlations across the data features, including six major hubs with higher expression in YST (LEFTY1, LEFTY2, miR302b, miR302a, miR 126, and miR 122) compared with other GCT.

**Conclusions:**

While prognosis for GCTs is overall favorable, many patients experience resistance to chemotherapy, relapse and/or long term adverse health effects following treatment. Targeted therapies, based on integrated analyses of molecular tumor data such as that presented here, may provide a way to secure high cure rates while reducing unintended health consequences.

## Background

Germ cell tumors (GCTs) include germinomas, comprised of testicular seminomas and ovarian dysgerminomas, and nonseminomas, comprised of yolk sac tumors (YSTs), teratomas and embryonal carcinoma [1]. While GCTs are heterogeneous, they are grouped together due to a presumed common stem cell of origin, the primordial germ cell (PGC). Oosterhuis and Looijenga have proposed classification of GCTs into five distinct entities based on cell of origin, histology, genomic imprinting status, age at and location of clinical presentation, and chromosomal constitution [2]. Type I GCTs are those found predominantly in infants and young children, often manifesting in the first four years of life and always before puberty. Type II GCTs are most commonly found in the testis of adolescent males and young men following puberty, but are also found in the ovaries of adolescent and young adult women and the midline/brain of children and adolescents. Pathologic evidence confirms that GCTs are the neoplastic counterpart of the PGC [2], and several lines of evidence indicate that GCTs, including adult testicular GCT (TGCT), begin *in utero* [3]. Thus, alterations in normal embryonic development are likely to be etiologically relevant to GCTs. Of particular interest are the processes the PGCs undergo during normal development, including segregation from the somatic cells, migration to the gonads, complete epigenetic reprogramming, reacquisition of pluripotency and sex determination [4].

Aberrant DNA methylation has been implicated in cancer etiology, and may be especially relevant in GCTs due to the extensive epigenetic reprogramming that occurs in the germ line and early embryo during normal development [5]. Adult TGCTs have been studied most thoroughly in the context of DNA methylation, and thus a majority of our knowledge regarding methylation is limited to these tumors. Interestingly, methylation patterns in GCT differ by histologic subtype in both adults and children [6–16]. In general, methylation increases with tumor differentiation: the lowest levels of methylation occur in the embryonal carcinomas and the highest in the teratomas [6, 7, 10, 11, 13, 15–18]. Understanding methylation patterns in GCTs, overall and by histologic type, may identify the developmental stage at which the tumor arose. This knowledge in turn may identify the at-risk period when external exposures are most harmful.

MicroRNAs (miRNAs) are small endogenous noncoding RNAs that regulate gene function in a manner specific to cell type and developmental stage [19–23]. Differential miRNA expression is associated with human cancers [24–28], including GCTs in children and adults [29–35]. These studies have reported higher expression of miRNAs in the miR-371–73 and the miR-302 clusters and lower expression of let-7 in Type I and Type II GCTs compared to normal samples [29–37]. Alterations in the serum levels of the miR371–3 and miR-302/367 MiRNAs also show promise as a diagnostic and follow-up tool for TGCT patients [38], highlighting the potential translational impact of molecular evaluation.

Knowledge of stem cell biology is directly relevant to mechanisms of GCT tumor initiation, maintenance and metastasis, since reacquisition of pluripotency is a key step in early germ cell development [39]. Typically, expression of stem cell markers (e.g., OCT3/4, STELLAR, NANOG, LIN28) is induced following demethylation of early stage germ cells [6, 17] and is turned off following entry to meiosis [40–42]. Expression of pluripotency markers past the appropriate developmental stage is a hypothesized explanation for tumorigenesis in germ cells [41]. Notably, studies of adult TGCT have shown aberrant expression of stem cell markers in intratubular germ cell neoplasia (IGCNU), the precursor of TGCT, and in undifferentiated histologic subtypes of GCTs (seminomas and embryonal carcinomas) [43, 44]. Stem cell markers are also expressed in early germ cells in females [45–47] and have been detected in ovarian dysgerminomas [48]. Marker expression past the appropriate developmental stage is correlated with genetic variation, including mutation in *c-KIT* [48] and its ligand (*KITLG*) [49], and DNA methylation [41]. Given that pediatric GCTs likely originate from a germ cell at an earlier stage of development than adult TGCTs [2], stem cell marker expression may be particularly relevant in pediatric tumors.

As described above, previous studies have described variation in methylation, miRNA, and mRNA expression in Type I and Type II GCTs, including studies that have evaluated the interaction between miRNA and mRNA expression [29, 35]. To further explore relevant molecular interactions, we used an integrated approach to understand differences in promoter methylation, miRNA expression, stem cell gene expression, and genotype data by tumor characteristics in a series of GCTs. We evaluated correlations between data based on the assumption that these processes are linked and co-regulated (for example, epigenetic changes within promoter regions and expression of cognate miRNA species determine the level of mRNA). We also find differences in miRNA expression and stem cell gene expression by tumor histology.

## Methods

### Study samples

Type I and Type II GCT samples from males and females were obtained from the Cooperative Human Tissue Network (Columbus, OH). Tumors were resected at initial diagnosis and snap frozen at −70 °C. Pathology reports were also provided. Data were available for tumor histology (YST, teratoma, germinoma, or mixed/other), tumor location (gonadal or extragonadal), sex, and age at diagnosis (< 10 years, ≥ 10 years). The age categories were chosen based on tumor histology. The majority of the tumors diagnosed between the ages of 4 and 10 were of similar histology to the Type I tumors while tumors diagnosed after age 10 included histologic subtypes typically included in the Type II category. Normal adjacent tissue was also available for seven of the tumors in our case series.

This analysis used existing data with no personal identifiers; therefore, the study was deemed exempt from review (category #4) by the Institutional Review Board of the University of Minnesota.

### DNA and RNA extraction

Genomic DNA was isolated from GCT tissue and paired normal adjacent tissue (when available) using either the TRIzol® extraction method (Invitrogen Life Technologies, California) or a QIAamp DNA Mini Kit (Qiagen Sciences, Maryland) according to the manufacturer’s recommended protocol. DNA yield was quantified using 1 μl DNA on a NanoDrop™ spectrophotometer (Thermo Scientific, Maryland). Extracted DNA was stored at −80 °C until further analysis.

Total RNA was extracted from fresh frozen tissue using the TRIzol® extraction method (Invitrogen Life Technologies, California) according to the manufacturer's protocol. Following extraction, RNA was cleaned using the RNeasy Mini Kit (Qiagen, Maryland) according to the manufacturer's recommended protocol. RNA yield was then quantified using 2 μl on a NanoDrop™ spectrophotometer (Thermo Scientific, Maryland). Extracted RNA was stored at -80^O^ C until further analysis.

### Methylation analysis

DNA methylation was measured as previously described [16]. Briefly, prior to methylation analysis, 1 μg genomic DNA was treated with sodium bisulfite to convert unmethylated cytosines to uracil using the EZ DNA Methylation Kit (Zymo Research, Orange, CA) according to manufacturer’s protocol. DNA methylation at 1505 CpG loci in 807 cancer-related genes was evaluated using the GoldenGate Cancer Methylation Panel I (Illumina, Inc.) in the University of Minnesota Genomics Center following the manufacturer’s protocol as described [50]. Replicates were included, including four duplicates that were included on both arrays and five duplicates that were included within one array.

Methylation was calculated as the variable β, which is the ratio of the fluorescent signal from the methylated allele to the sum of the fluorescent signals of both methylated and unmethylated alleles [50]. GenomeStudio software (Illumina, Inc) was used to calculate the average methylation values (β) from the ~30 replicate methylation measurements for each CpG locus. We used raw average β values without normalization. GenomeStudio software was also used to assess data quality for each CpG loci. We omitted all CpG loci where ≥ 25 % of the samples had a detection *p*-value > 0.05 (*N* = 16, 1 %). X-linked CpG loci (*N* = 84) were also removed, resulting in 1,405 loci for analysis.

### miRNA expression

Expression of 800 miRNAs was measured using the NanoString nCounter miRNA Expression Assay kit (NanoString Technologies, Seattle, WA). The nCounter detects total counts of miRNA through hybridization with fluorescently labeled bar coded probes to the miRNAs of interest followed by scanning and counting to quantify expression [51]. Total RNA samples were analyzed following the manufacturer’s instructions for the Human v2 miRNA Expression Assay Kit (NanoString Technologies, Seattle, WA).

### Stem cell factor expression

Real-time quantitative PCR gene expression profiling was performed for 84 pathway-specific genes using the human Embryonic Stem Cells RT^2^ Profiler PCR Array according to the manufacturer’s protocol (SABiosciences, Frederick, MD). Briefly, the RT^2^ First Strand Kit (SABioscience, Frederick, MD) was used to synthesize cDNA from 1 μg purified RNA. cDNA was obtained with a High Capacity cDNA Reverse Transcription Kit (Applied Biosystems, Foster City, CA), and quantitative PCR (qPCR) was performed with a StepOnePlus Real-Time PCR system (Applied Biosystems, Foster City, CA). Expression of all genes was normalized to average expression of five endogenous housekeeping genes (E2M, HPRT1, RPL13A, GAPDH, and ACTB).

### Genotyping

Genotype data were generated for four SNPs identified in GWAS of adult TGCT as previously described [52]. Briefly, PCR amplification and sequencing were performed for SNPs in three genes: *SPRY4* (rs4324715), *BAK1* (rs210138) and *DMRT1* (rs755383). The *KITLG* (rs4474514) SNP was detected using a made-to-order TaqMan® SNP Genotyping Assay from Applied Biosystems Inc (catalog# 4351379, assay# C_26154778_10). Primers and conditions for all assays are available upon request.

### Statistical analysis

#### miRNA analysis

We used NanoStriDE for normalization and differential expression analysis of the miRNA data [53]. Positive control normalization was conducted by creating a normalization factor for each sample using the 6 positive assay controls on the array and negative control normalization was conducted as an upper quantile approach as recommended by the manufacturer (NanoString Technologies, Seattle, WA). Discrete count data were compared across demographic and tumor characteristics using a negative binomial distribution as described by Anders and Huber [54]. We included a Benjamini-Hochberg correction for multiple comparisons [55].

#### Stem cell factor expression analysis

Raw gene expression values were normalized to endogenous housekeeping genes prior to statistical analyses. Unsupervised hierarchical clustering was conducted using the matrix visualization and analysis platform Gene-E with the city block metric and average linkage [56]. Fold change of gene expression was determined using the 2^(−ΔΔCt)^ method, and compared YST (*n* = 9) to germinomas (*n* = 8).

#### Cross platform analysis

Patient age and sex, tumor location (ovary, testis, and extragonadal), and histology (normal adjacent, teratoma, dysgerminoma, YST and mixed) data were combined with molecular data across a common set of 40 samples into a single two-dimensional matrix. Categorical phenotype and genotype values were arranged in a logical manner (e.g., low severity to high severity for tumor histology) and assigned a numerical code that could be used for correlation analysis. These data were combined with the β values from the methylation analysis, the miRNA counts, and the normalized ΔCt values from the SABiosciences Embryonic Stem Cell array. Additionally, genotypes for the four molecular markers listed above were encoded based on the Kimura matrix [57] idea that transitions are more likely than transversions and hence grouped together where possible.: −4.0 = t/t; −3.0 = c/t; −2.0 = c/c; −0.5 = g/t; −0.2 = c/g; 0.2 = a/t; 0.5 = a/c; 2.0 = a/a; 3.0 = a/g; 4.0 = g/g.

Two types of correlations were explored using the vector of values for each matrix row (i.e., molecular probe or phenotypic label) against the corresponding vector of values for each of the other rows: standard linear correlations using the Pearson correlation coefficient and more complex nonlinear correlations using the Maximal Information Coefficient (MIC) [58]. MIC analysis was performed using the R implementation of the MINE software package (http://www.exploredata.net/). This resulted in two values for correlation (linear, nonlinear) for every pairwise combination of molecular probes and/or phenotypic variables. This very large result matrix was filtered to retain only those pairs that represented a comparison across platforms (or between a phenotypic variable and a platform) and whose Pearson correlation ***or*** MIC values exceeded a threshold of 0.75. This threshold was chosen as it provided a compromise between very dense graph connectivity at lower thresholds and sparse connectivity at high cutoffs.

The resulting pairwise correlations were visualized as a network using Gephi software (https://gephi.org/). In order to visualize the correlation networks, the molecular probes and phenotypic variables served as nodes in the network graph, and an edge was drawn between any nodes that had either a linear or nonlinear correlation that exceeded 0.75. The edge was labeled with the larger of either the Pearson *R*-value or the MIC. These data were loaded into Gephi, and the Force Atlas 2 layout was executed, running until the nodes were far separated in apparent equilibrium, and then the Fruchterman Reingold layout was selected. All pairwise correlations were grouped by single linkage clustering to create networks. Network hubs with four or more neighbors were identified.

The hub nodes and their nearest neighbors were concatenated into a list, and the standard gene symbols extracted. These gene symbols were uploaded to Ingenuity Pathway Analysis software (IPA, http://www.ingenuity.com/products/ipa), and investigated for upstream activators. To guard against the possibility that functional categories were identified by chance due to the *a priori* bias in the initial set of profiling molecules, we randomly selected three additional sets of the same size from the same initial profiling molecules, and submitted these random gene lists to IPA.

The entire analysis process was repeated using subsets of the data. In the entire data set of 40 samples with complete genomic data, it was noted via manual analysis of scatterplots of primary hub genes that differences in the 8 YSTs relative to the other samples dominated the signal. Following the assumption that the YSTs were overshadowing any signal from other histology types, we tested a subset consisting of the 32 non-YST samples.

## Results

### Characteristics of the study samples

Tumor specimens from 38 cases of Type I and Type II GCT ranging in age from 0 to 21 years were included in this analysis, including 9 YSTs, 18 teratomas, 7 dysgerminomas, and 4 with mixed histology (Table 1). In addition, one dysgerminoma from a 45 year old woman (Type II GCT) was included because it had similar methylation, miRNA and stem cell factor expression values as the adolescent dysgerminoma samples. The YSTs were evenly distributed among boys and girls while the majority of cases with a germinoma or teratoma were female. Information on race/ethnicity was not available for the cases.

**Table 1:** Selected characteristics of the study population

	Yolk Sac Tumor	Dysgerminoma	Teratoma	Mixed/Other	Normal
	*N* (%)	*N* (%)	*N* (%)	*N* (%)	*N* (%)
Age (years)					
Median (range)	1 (0–19)	13.5 (7–45)	4.5 (0–21)	10 (0–14)	11 (1–19)
Sex					
Male	5 (56)	0	3 (17)	3 (75)	2 (29)
Female	4 (44)	8 (100)	15 (83)	1 (25)	5 (71)
Location					
Ovary	2 (22)	8 (100)	9 (50)	1 (25)	1 (14)
Testis	2 (22)	0	1 (5.6)	2 (50)	1 (14)
Extragonadal	5 (56)	0	8 (42)	1 (25)	5 (71)

The four cases with mixed histology all had a teratoma component of the tumor. The three tumors in male cases also had YST. One mixed tumor in an adolescent male and the tumor in an adolescent female also had components of embryonal carcinoma and choriocarcinoma. Normal adjacent DNA was available for seven cases. The seven normal adjacent tissue samples included four ovary or fallopian tube tissues, one testis tissue, one adjacent lymph node and one thymus tissue. The testis sample was from a one year old case and is unlikely to contain IGCNU. Results from methylation and genotyping data have been previously published [16, 52]. and are therefore not included in this report.

### miRNA expression

**Fig. 1:**
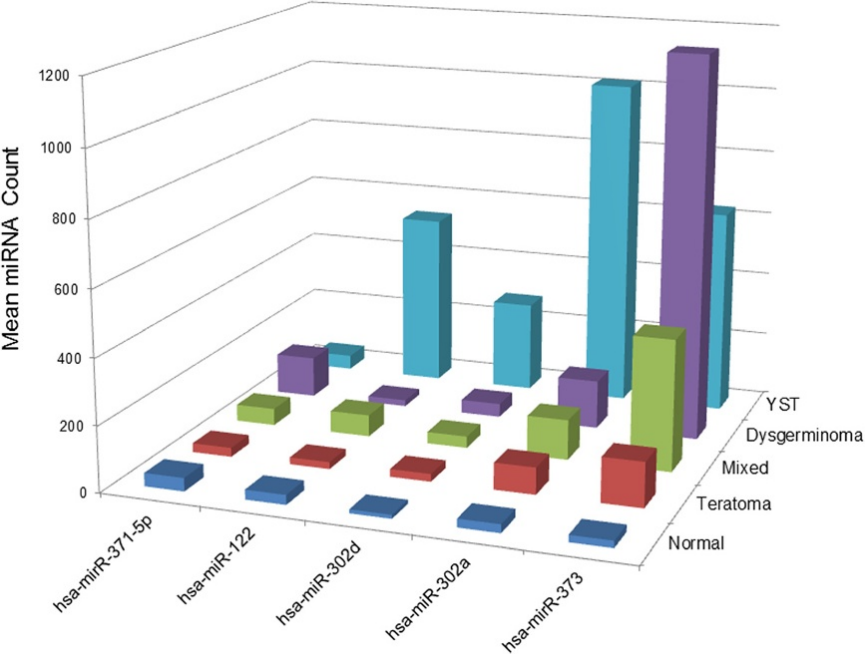
miRNA expression by tumor histology. Five miRNA species had significant expression differences by tumor histology (*q*-value < 0.05). Three samples were excluded due to missing or poor quality miRNA data (1 YST, 1 mixed/other, and 1 normal adjacent)

In a comparison across all histologic subtypes, we observed significant differences in miRNA expression for five miRNA species (Fig. 1). Of these, all had low expression in normal adjacent tissue. One miRNA was elevated only in dysgerminomas (hsa-miR-371–5p). The other four miRNAs were elevated in YST with varying expression differences in other histologic subtypes. We did not observe differential expression for any miRNA species when we compared gonadal vs. extragonadal tumors, tumors from males vs. females, or tumors from children diagnosed prior to age 10 years vs. those diagnosed at or after 10 years of age (data not shown).

### Stem cell factor expression

**Fig. 2:**
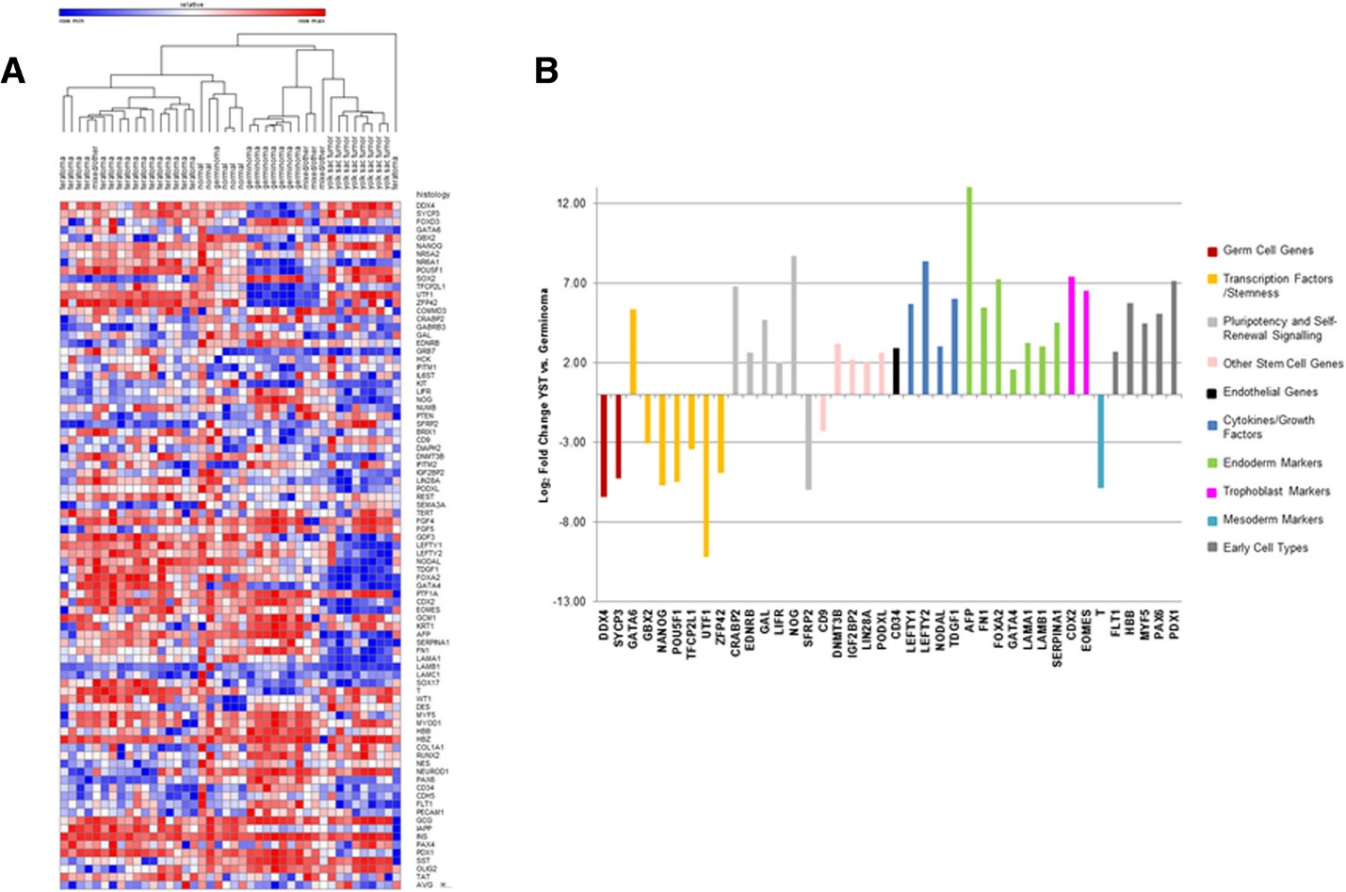
Stem cell expression by tumor histology. **a** Unsupervised hierarchical clustering analysis of normalized ΔCt values. Blue indicates high levels of expression and red indicates low levels of expression. The mixed tumor that clustered with the teratomas rather than the other mixed tumors included components of teratoma and YST. **b** Genes with ≥ 3 fold (log_2_ fold > 1.58) up- or down-regulation in YST compared with germinoma. Bar color represents gene pathway. Four samples were excluded due to missing data (1 YST, 1 teratoma, and 2 normal adjacent)

Unsupervised hierarchical clustering based on tumor histology highlights differences in the expression of stem cell genes by tumor histology (Fig. 2a), with the YST and dysgerminoma separating into distinct clusters, while the teratomas (mature and immature) and normal adjacent tissue clustered together. Mixed GCTs were interspersed throughout the groups. To better understand the expression differences in YST and dysgerminoma, we compared relative expression of each gene in these two groups. Of the 84 genes included in the array, 40 had a statistically significant up- or down-regulation in YST compared with dysgerminoma (*p* < 0.05). When these genes were categorized by function in pathways regulating initiation and maintenance of cellular stemness, we noted distinct patterns based on tumor histology (Fig. 2b). For example, transcription factors related to stemness had higher expression in dysgerminomas, while endoderm, trophoblast, and mesoderm markers had higher expression in YST.

### Cross-platform Analysis

**Fig. 3:**
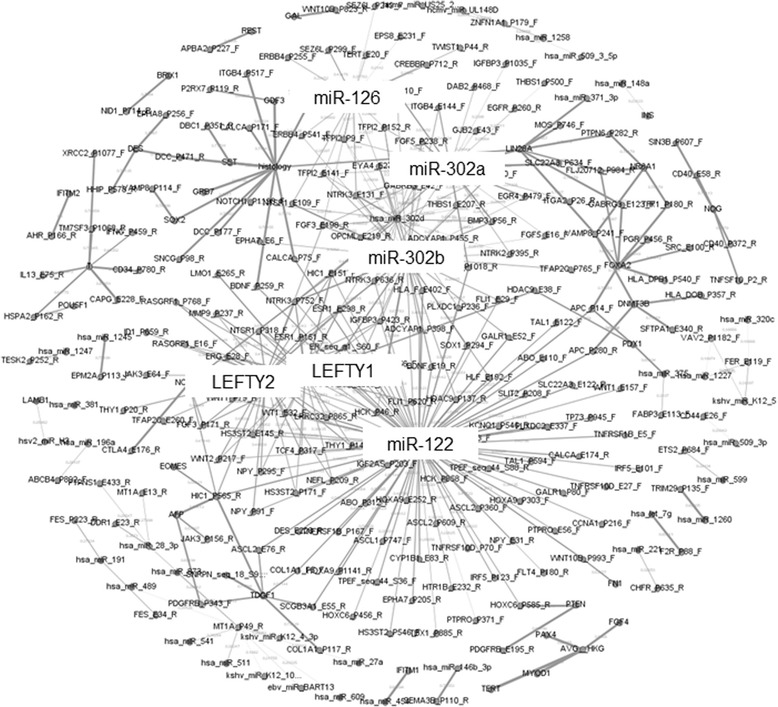
Visualization of cross-platform correlations using Gephi. 760 cross-platform correlations had a Pearson correlation or a MIC that exceeded a threshold of 0.75. Network visualization via Gephi identified five network hubs (>4 nearest neighbors) that differentiated YST from the other histologic subtypes. Seven samples were excluded because they did not have complete data for methylation, miRNA, stem cell gene expression (2 YST, 1 teratoma, 1 mixed/other, and 3 normal adjacent)

We observed 760 cross-platform correlations that exceeding our threshold of 0.75 for either the Pearson correlation coefficient or the MIC. When these data correlations were visualized, six network hubs were identified (Fig. 3), including the miRNAs, miR-122, −126, −302a and -302b and the stem cell genes, LEFTY1 and LEFTY2. These changes were influenced largely by differences in the expression in YST vs. tumors of other histologic subtypes. In order to detect differences that may have been overshadowed by the strong influence of the YSTs, we also repeated the analysis including only the dysgerminomas and teratomas. In this analysis, the protein T (brachyury) was identified as a network hub linking a large number of molecular features (data not shown).

Using IPA, we identified many predicted upstream activators that were enriched in the list of genes with large cross platform correlations (*N* = 45 genes). After comparison with the *p*-values from the randomly selected gene lists, there were three molecules (TP73, decitabine, and tretinoin) with statistically significant *p*-values after Benjamini-Hochberg correction, suggesting that these molecules are promising upstream activators.

## Discussion

In this analysis, we used a novel method to integrate molecular data across platforms to gain biological insight into the function of GCTs. These analyses highlighted several network hubs, including miRNA clusters and stem cell genes that distinguish YST from normal germ cell samples and other GCTs. Importantly, our approach confirmed several previously reported alterations in embryonic stem cell specific miRNAs. Finally, our analysis of stem cell factor expression highlights the altered expression of multiple stem cell genes in GCTs, with distinct patterns in YST and dysgerminoma. Collectively, these findings suggest that ectopic, aberrant expression of stem cell genes may underlie the unusual and defining capacity of self-renewal in the face of wide differentiation into cells with characteristics of tissues derived from any of the three germ layers observed in GCT, and it is possible that higher levels of stem cell gene expression correlate with tumor progression and prognosis of GCT and other tumors.

Our findings are consistent with previous studies of Type I and Type II GCTs that have identified an overexpression of the miR-371–73 and miR-302 clusters in GCTs compared with normal samples [29–35]. The miRNA-302 and miRNA-371–373 clusters are highly plausible candidate miRNAs in GCTs given their roles as regulators of embryonic stem cell pluripotency markers [59, 60] which has been discussed extensively in previous miRNA expression studies of GCTs [33, 35]. Pinpointing the relevant targets of miRNA can be daunting given the large number of target proteins for each miRNA. According to the online database for miRNA target prediction and functional annotations, miRDB [61, 62] the number of predicted targets for miRNAs in the miR-371–373 and miR-302 families range from 469 to 529; interestingly, LATS2 is one of the highest ranking targets on the list for all miR-302 family members as well as miR-372 and miR-373 (Target Score > 98) [62]. Functional studies have demonstrated that miRNA-372 and miRNA-373 act as oncogenes in TGCT through interactions with the p53 pathway, in particular through regulation of LATS2 [32]. Bioinformatic algorithms indicated that miRNA expression in these clusters is associated with downregulation of mRNA expression in other pathways with biological relevance to GCT [33, 35]. Data also suggest that the miRNA 302 family can be used to reprogram cancer cells into pluripotent cells with an ES cell-like phenotype [63]. Collectively, these data suggest that these miRNA clusters are in large part responsible for regulating the stem cell phenotype of GCTs.

Four of the six hubs identified in the cross-platform analysis were stem cell related, and highlight the importance of the transforming growth factor (TGF)-β superfamily members in GCT development. Specifically, the miR-302 cluster regulates the Nodal inhibitors LEFTY1 and LEFTY2 [64]. This interaction plays an important role in germ layer specification by promoting the formation of the mesendodermal lineage while suppressing neuroectorderm formation [65]. Knockdown of either protein in mice results in altered differentiation, with *Lefty1* knockdown leading to increased differentiation potential and *Lefty2* knockdown leading to increased immature neuroepithelium [66]. This pathway also plays other important roles in germ cell development, including regulating meiosis in the male germ cells [67, 68]. Finally, the nodal signaling pathway regulates the bone morphogenic protein pluripotency pathway [69], which was previously shown to play an important role in the development of GCT [70]. The importance of miR-122 and miR-126 in GCT is less clear and will require further study. Interestingly, miR-122 was also overexpressed in YST compared with germinoma and normal gonad tissue in a previous study of miRNA in GCT [33]. Notably, none of the methylation differences that were so striking in our comparison of YST to other GCTs [16] were identified as being hubs in the cross-platform analysis, suggesting that these changes may be consequence of the altered expression of stem cell genes and miRNAs rather than drivers of the oncogenic process. Given that the associations between the four SNPs evaluated did not differ by tumor histology [52, 71, 72], it was not as surprising that none of these were identified as hubs in the comparison of tumors by histology.

We chose to use platforms that were highly enriched for cancer and stem cell genes due to the higher information yield when compared to an unbiased search; however, this limited our ability to conduct an unbiased search for over-represented gene categories in the list of features with high cross-platform correlations. We were able to evaluate upstream regulators of these highly correlated features, and we identified three highly enriched activators (TP73, Tretinoin, and Decitabine) in the set of hub-connected genes in the entire dataset. Given the known importance of retinoic acid in germ cell development [73], it is not surprising that Tretinoin, a topical retinoid, would be identified as a potential regulator in GCTs. The DNA hypomethylating agent Decitabine (5-aza-2’-deoxycytidine or 5-aza) is also intriguing as a potential therapy given relevant data in the literature. Decitabine is effective for treatment of hematologic malignancies [74, 75] and has also been evaluated in studies of solid tumors with varying success rates [76]. Preclinical data suggesting that hypomethylating agents may re-sensitize cells to platinum based chemotherapy [77–79] are of particular relevance for GCT; however, early phase clinical trials in epithelial ovarian cancer have provided mixed results of the combination of decitabine and carboplatinum [80–82]. In a previous study of embryonal carcinoma, high expression of the pluripotency-associated DNA methyltransferase 3B (DNMT3B) was associated with sensitivity to 5-Aza [77, 83]. The elevated expression of DNMT3B in YST observed here and in a previous DNA methylation study [15] suggests that this may also be a relevant alternative therapy for YST, which often have poorer outcomes than other histologic subtypes [84].

Most cancers are heterogeneous and multifactorial [85]. This complexity stems from molecular events on the level of gene integrity, epigenetic modification and transcription, stability of mRNA transcripts (e.g., by miRNAs), translation, protein activation (e.g., by phosphorylation), and cellular interactions, including within the tumor microenvironment. Because of this complexity, it is important to evaluate the joint effects of these alterations. There is not one clear method for evaluating these complex interactions on large datasets. In this analysis, we chose to utilize the MIC as a tool to evaluate the correlations in the dataset in addition to simple linear Pearson correlation. MIC is able to detect linear relationships and correlations among variables that are not strictly linear (such as quadratic or oscillatory associations). Care should be taken when using the MIC with small datasets (e.g., *N* < 30), as it may identify spurious correlations in this extreme [86]. Also, despite a recent debate [87] as to which information-theory based measure provides the highest statistical power (maximal information coefficient or a related measure, mutual information), both measures clearly identify nonlinear trends that are missed by Pearson correlation. Additionally, our analysis included a small number of samples which could have limited our power to detect relevant associations. Finally, our analysis focused on a selected list of genes with a priori significance in cancer and stem cells. It is possible that a genome-wide, agnostic approach would identify additional relevant characteristics of GCTs in additional pathways.

## Conclusions

Our analysis suggests that the stem cell phenotype of GCTs is a defining characteristic of GCTs, especially in YSTs. While prognosis for GCTs is overall favorable, subgroups of patients experience resistance to chemotherapy and/or high rates of relapse [88–90]. In addition, the nonspecific and highly cytotoxic chemotherapy used can cause many adverse health effects including cardiovascular disease, hearing loss, and second cancers [91–95]. Targeted therapies, based on integrated analyses of molecular tumor data such as that presented here, may provide a way to improve cure rates in the subgroup of patients who fail to respond to current therapies and may also provide an opportunity to reduce the unintended health consequences associated with current chemotherapeutic agents.

## Abbreviations

GCT: Germ cell tumor
YST: Yolk sac tumor
MIC: Maximum information coefficient
PGC: Primordial germ cell
TGCT: testicular germ cell tumor
IPA: Ingenuity Pathway Analysis
TGF: Transforming growth factor
DNMT3B: DNA methyltransferase 3B
